# Epigenetic Regulation by Non-Coding RNAs in the Avian Immune System

**DOI:** 10.3390/life10080148

**Published:** 2020-08-12

**Authors:** Xiaolan Chen, Bahareldin Ali Abdalla, Zhenhui Li, Qinghua Nie

**Affiliations:** 1 Lingnan Guangdong Laboratory of Modern Agriculture, College of Animal Science, South China Agricultural University, Guangzhou 510642, China; xiaolanchen@stu.scau.edu.cn (X.C.); abdalla406@163.com (B.A.A.); 2 Guangdong Provincial Key Lab of Agro-Animal Genomics and Molecular Breeding, and Key Laboratory of Chicken Genetics, Breeding and Reproduction, Ministry of Agriculture, Guangzhou 510642, China

**Keywords:** non-coding RNAs, long non-coding RNAs, circular RNAs, microRNAs, avian diseases

## Abstract

The identified non-coding RNAs (ncRNAs) include circular RNAs, long non-coding RNAs, microRNAs, ribosomal RNAs, small interfering RNAs, small nuclear RNAs, piwi-interacting RNAs, and transfer RNAs, etc. Among them, long non-coding RNAs, circular RNAs, and microRNAs are regulatory RNAs that have different functional mechanisms and were extensively participated in various biological processes. Numerous research studies have found that circular RNAs, long non-coding RNAs, and microRNAs played their important roles in avian immune system during the infection of parasites, virus, or bacterium. Here, we specifically review and expand this knowledge with current advances of circular RNAs, long non-coding RNAs, and microRNAs in the regulation of different avian diseases and discuss their functional mechanisms in response to avian diseases.

## 1. Introduction

About 1% of the mammalian genome has the potential to encode proteins, which means a huge amount of non-coding RNAs (ncRNAs) exist in mammalian transcripts [1]. The huge amount of ncRNAs indicated that they are very important biological molecules in epigenetic regulation. Although the current studies on ncRNAs are not elaborate, its important functions for various biological processes have been discovered, especially its extensive roles in different kind of diseases. As a pillar of the economy and people’s livelihood, poultry industry becomes important for our life, the meat and egg of chicken is directly associated with the human health. But the threat of disease infection to poultry farming has never been absent, which means it requested from us to develop the poultry host immunity to prevent the infection of avian diseases. Many factors have an impact on the chicken innate and acquired immunity, such as environment, nutrition, and genetics. As the most complicated factor, genetics has its special power to improve the host defense. Genetic improvement is the most effective strategy to deal with disease invasion, and is a complex biological process of multilevel regulation, in which ncRNAs played their unique role. Numerous studies have been conducted on ncRNAs analysis in avian diseases; therefore, summarizing the roles of ncRNAs in the regulation of avian immunity is helpful for us to investigate the new potential function of ncRNAs in avian immunity.

The classical recognition of ncRNAs is that they are the products of genome DNA transcription but they lack the ability to encode a functional protein [2]. However, with the continuous development of the technology of high-throughput sequencing, the implementation of ribosome sequencing (Ribo-Seq) has given the evidence that most of the ncRNAs can be bound to ribosome, especially the large intergenic noncoding RNA (lincRNA) [2]. Few percentages of those ribosome-associated ncRNAs have small open reading frames (sORFs) [3,4] and have the ability to encode one or more protein that is usually no more than 100 amino acids [3,5]. It means that ncRNAs are not absolutely unencoded. Even through, some ncRNAs have the ability to encode small peptide, the majority of the ncRNAs do not function through encoded protein [2]. They are still called non-coding RNAs. ncRNAs includes: long non-coding RNAs (lncRNAs), circular RNAs (circRNAs), microRNAs (miRNAs), ribosomal RNAs (rRNAs), small interfering RNAs (siRNAs), small nuclear RNAs (small nuclear RNAs), piwi-interacting RNAs (piRNAs), and transfer RNAs (tRNAs), etc., [6,7,8]. The well-known ncRNAs, such as lncRNA, circRNA, and miRNA, were selected here to summarize their functional mechanisms and their important roles in avian immune system.

## 2. The Specific Roles of LncRNA, Circular RNA, and miRNA

**Figure 1:**
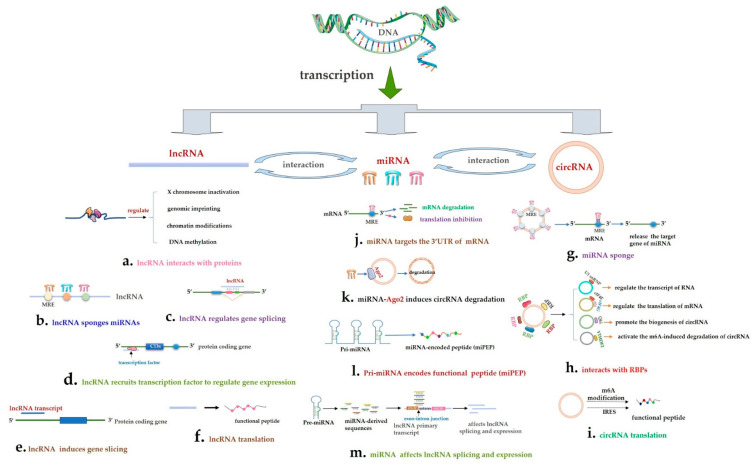
The functional mechanism of lncRNAs, circRNAs, and miRNAs. (**a**) LncRNA interacts with proteins. (**b**) lncRNA acts as a miRNA sponge. (**c**) LncRNA regulates gene splicing. (**d**) LncRNA recruits transcription factor to regulate gene expression. (**e**) LncRNA transcriptional overlap, but not its lncRNA products, induces gene slicing. (**f**) LncRNA translation. (**g**) circRNA acts as miRNA sponge. (**h**) circRNA interacts with RNA binding proteins (RBPs). (**i**) circRNA translation. (**j**) miRNA targets the 3′UTR of the mRNA. (**k**) miRNA-Ago2 induces circRNA degradation. (**l**) Pri-miRNA encodes functional peptide. (**m**) pre-miRNA affects lncRNA splicing and expression.

LncRNAs are linear non-coding RNAs that are more than 200 bp in length [1]. It is known that lncRNA can arise from various transcript products of genome, such as intronic transcript, antisense transcript, intergenic transcript, enhancer, pseudogene, and retrotransposon [2,9]. So according to the relative position of the lncRNA and the encoded gene, there are antisense lncRNAs, divergent lncRNAs, intron lncRNAs, intergenic lncRNAs, enhancer-associated lncRNAs [10], promoter-associated lncRNAs [10], and transcription start site-associated lncRNAs in the cluster of lncRNAs. Normally, lncRNAs act as cis-acting elements or trans-acting factors to regulate gene expression at pre-transcriptional, transcriptional, and post transcriptional processing. LncRNA can interact with different proteins (Figure 1a) to participate in X chromosome inactivation [11], genomic imprinting [1,12,13,14], chromatin modifications [15,16,17], DNA methylation [18,19,20], mRNA degradation [21,22]. Additionally, lncRNA can act as a competing endogenous RNA (ceRNA) of miRNA and sponge miRNA (Figure 1b) to release the inhibition of miRNA to their target genes. It also regulates mRNA splicing (Figure 1c) [23]. LncRNAs regulate gene splicing (Figure 1d) by transcriptional interference [24]. Noncanonically, some lncRNAs have sORFs and exhibit their special regulatory functions through coding one or more micro-peptide (Figure 1f) [4].

Circular RNAs are special endogenous ncRNA molecules that contain a closed loop structure. Previously, we knew that the majority of identified circRNAs were from only exonic region or only intronic region or both exonic and intronic region, and relatively fewer circRNAs produced by intergenic regions, antisense transcripts of known transcripts, untranslated regions (UTRs), and other regions in eukaryotes [25]. So circRNAs are mainly classified in exonic circular RNA (eciRNA), intronic circular RNA (ciRNA), and exon-intron circular RNA (EIciRNA). But recently, circRNAs from mitochondria DNA were newly investigated, this kind of circRNA was called mecciRNAs [26]. Because circRNAs have no free 5′cap and 3′ poly A tail, it makes them not easily degraded by RNase. Sometimes, they are more stable than other RNAs and even expressed more abundantly than parental gene in eukaryotes [27]. As a kind of stably existing RNA, several circRNA functional mechanisms have been investigated, such as acting as miRNA sponges [28] (Figure 1g), interacting with RNA binding protein (RBP) [29,30] (Figure 1h) and translating to a functional peptide (Figure 1i) [31,32]. Among them, circRNA as miRNA sponge is the most well studied. It is presented that circRNAs have a certain amount of miRNA biding cites and could arrest miRNA and then release miRNA targets [33]. The interaction with RBP makes circRNAs possess various regulatory functions. For instance, circRNA interacts with small nuclear ribonucleoprotein U1 subunit (U1 snRNP) in the nucleus to regulate the transcript of RNA [34]; circRNA binds to *eukaryotic initiation factor 4E (eIF4E)* and *eukaryotic initiation factor 4G (eIF4G)* to regulate the translation of mRNA [29]; quaking (QKI) protein interacts with the pre-mRNA of gene and promotes the biogenesis of circRNA [35]; methylation-related protein-YTH N6-methyladenosine RNA binding protein 2 (YTHDF2) interacts with circRNA and activates the m(6)A-induced degradation of circRNA [30]. As the most novel functional mechanism, circRNA translation was mainly discovered in circRNA which has internal ribosome entry site (IRES) or m6A modification [36]. IRES allows direct recruitment of initiation factors and ribosomes on the RNA and initiates the circRNA translation through cap-independent manner [37,38]. It is discovered that some endogenous circRNAs that have m (6) A motifs have the ability to translate and the abundance of m (6) A motifs could affect the translation efficiency of circRNA [36]. However, it requires the involvement of *eukaryotic translation initiation factor 4 gamma 2* (*eIF4G2)* and *m (6) A reader YTH N6-methyladenosine RNA binding protein 3* (*YTHDF3)* and will be affected by other m (6) A modification-related proteins. It can be enhanced by *methyltransferase like 3/14* (*METTL3/14)* and suppressed by demethylase *FTO* [36].

miRNAs are a class of small ncRNAs that were ~22 nucleotides in length. The classical role of miRNA is binding to the 3′UTR (Figure 1j) of the mRNAs and then induce the degradation of mRNA or inhibit the translation of mRNA [39]. Notably, at the RNA world, miRNA plays a key role in the whole ncRNA regulatory network. It becomes the central of the ceRNA network and could bind lncRNAs/circRNAs with mRNAs as both circRNAs and lncRNAs have the miRNA response element (MRE site) and could act as a miRNA sponge [28]. LncRNA/circRNA-miRNA-mRNA axis is a powerful regulatory mechanism and was extensively investigated in various biological process of different species. With the deep study of molecular biology, miRNA was given several new functions. First, it could regulate the expression of circRNA by recruiting Argonaute (AGO) protein to interact with circRNA (Figure 1k). For instance, miR-671 regulates the production of a circ*CDR1* via cleaving the *CDR1* antisense transcript in an Ago2-mediated manner [40]. miRNA-1224 negatively regulates circRNA-Filip1l expression through binding and splicing pre-circRNA-Filip1l, which also requires the Ago2 protein [41]. Additionally, the m7G methylation of miRNA showed another functional mechanism of miRNA. It is evidenced that the m7G methylation of let-7 miRNA induced by *methyltransferase like 1 (METTL1)* could regulate the process of lung cancer [42]. Moreover, it was investigated that some pri-miRNAs in plants have the ability to encode a peptide (Figure 1l) that is termed as miRNA-encoded peptide (miPEP). The translation of the pri-miRNAs leads to the accumulation of their corresponding miRNAs, then causing the downregulation of miRNA targets. For instance, pri-miR-171b in *Medicago truncatula* (*M. truncatula*) contains two ORFs, ORF1 and ORF2; ORF1 could encode functional peptide, termed as miPEP171b. The overexpression of miPEP171b or the application of a synthetic miPEP171b could increase the abundance of miR-171b [43]. Similar functional mechanism was found in other pri-miRNAs, such as miR-160b, miR-164a, miR-169d, miR-171e, miR-319a, and miR-165a in *M.truncatula* or *Arabidopsis thaliana* (*A. thaliana*) [43,44]. Lastly, miRNA-derived sequences that are identical with pre-miRNAs could regulate lncRNA splicing through overlapping the pre-transcript of lncRNA (Figure 1m). Specifically, hsa-miR-99b regulates the splicing of the primary transcript of LINC01129 through complementary combination with its exon-intron junction [45]. In spite of the underlying mechanisms of those newly identified miRNA functions warrant further analysis, these novel functions provided us the new area to study the potential role of miRNAs.

The important roles of lncRNAs, circRNAs, and miRNAs in regulating the pathogenicity of many diseases have been mainly discovered in humans. The critical roles of ncRNAs in avian inflammation and autoimmune regulation were also investigated. This review sheds light on the regulatory mechanism of lncRNAs, circRNAs, and miRNAs on chicken immunity and be helpful for identifying potential ncRNA biomarkers for avian diseases

## 3. Invasive Diseases Causing Significant Economic Losses in Poultry Industry

Avian invasion diseases are mainly caused by parasites, viruses, or bacterial infections. They infect chickens by specific manners and cause serious damage to chicken organs, thus influencing livability and body health of chickens. The direct consequence of this damage is the decrease of egg and meat production, which can bring huge financial losses to the poultry industry. According to the research progress on ncRNAs, we selected the related diseases researches in avian to summarize here. Among them, virus infection diseases include Avian leukosis virus (ALV), Marek’s disease virus (MDV), infectious bursal disease virus (IBDV), Avian influenza virus (AIV), infectious bronchitis virus (IBV), Newcastle disease virus (NDV), and reticuloendotheliosis virus (REV). The epidemic parasitic diseases, coccidiosis and *Cryptosporidium baileyi* infections are selected to be focused here. The bacterial infection disease reviewed here includes *Campylobacter jejuni (C. jejuni)*, *Salmonella enterica* serovar Enteritidis (SE), and *Salmonella typhimurium (S. typhimurium)*.

## 4. The Role of ncRNAs in Virus-Induced Avian Disease

### 4.1. ncRNAs Involved in Avian Leukosis Virus

Avian leukosis virus (ALV) is a retrovirus belonging to a *retroviridae* family. ALV includes seven subgroups of A, B, C, D, E, J, and K, among which, ALV-J is the most pathogenic subgroup with high transmission and pathogenicity [46,47]. The clinical manifestation of ALV-J-infected chicken including pathogenic tumors, immune tolerance, delayed growth and also higher susceptibility to a secondary infection [46,48].

#### 4.1.1. circRNAs Involved in ALV-Infection

Several studies have been focused on the expression profile and the molecular regulatory mechanisms of circRNAs during the infection of ALV. Thousands of circRNAs were identified in various types of ALV-infected organs, and the number of differentially expressed circRNAs (DEcircRNAs) are calculated, ranges from dozens to thousands [49,50,51,52]. Gene ontology (GO) term and Kyoto Encyclopedia of Genes and Genomes (KEGG) pathway analysis showed that the DEcircRNAs were involved in immune-related GO items and KEGG pathways. Main GO terms include regulation of B-cell activation [49], B-cell differentiation [49,50], alpha-beta T-cell activation [49], and main KEGG pathways have signaling pathways of mTOR [49,50], TGF-beta [50], Toll-like receptor [50], RIG-I-like receptor [50], Jak-STAT [50], Insulin [50] and ErbB [50], MAPK [52], Wnt [52], and B-cell receptor [52]. Many circRNA–miRNA–mRNA ceRNA networks were characterized [49,50,51,52]. Even through so many circRNAs were identified, only few circRNAs were validated for their localization or functional mechanism. One exon-intron DEcircRNA, circHRH4 localizes in cytoplasm and was abundantly expressed in different chicken tissues and cells [50]. circ-Vav3 can interact with miR-375 and upregulate the expression of *yes-associated protein 1* (*YAP1*), a target gene of miR-375. circ-Vav3/miR-375/*YAP1* axis participated in tumorigenesis through activating epithelial-mesenchymal transition (EMT) by altering the expression of EMT markers, including *Vimentin*, *zinc finger E-box binding homeobox 1 (ZEB1)*, matrix metallopeptidase 2 (*MMP2)*, *N-cadherin*, *Fibronectin*, and *E-cadherin* [51].

As we know from the current research process, the role of circRNAs related to the infection of ALV largely remain on the early stage of the characterization, expression profile, and associated pathways. Only a few researches have explored the single circRNA molecular function in response to the ALV-infection, which inspired us to study the specific functional mechanism of single circRNA in regulating ALV-infection.

#### 4.1.2. LncRNAs Associated with ALV-Infection

Similar to circRNA, a few researches have been focused on the lncRNA expression during ALV infections. RNA-seq had been performed on both chicken tissues and cells to screen the ALV-associated lncRNAs. For instance, differentially expressed lncRNAs (DElncRNAs), differentially expressed miRNAs (DEmiRNAs), and differentially expressed mRNAs (DEmRNAs) between infected and non-infected tissues [53,54], chick embryo fibroblasts cells (CEF cells) [55] or chicken primary monocyte-derived macrophages (MDMs) [56], were identified. Some important immune-related pathways were enriched, including Toll-like receptor, NODlike receptor, RIG-I receptor, and JAK-STAT signaling pathways [55]. The co-expression network analysis among those DEmRNAs, DEmiRNAs, and DElncRNAs revealed that the DElncRNAs could interact with immune-related miRNAs and genes to exhibit their role in diseases and cancers process [53,54,55,56]. Specially, some lncRNA-miRNA-mRNA interaction networks participated in the regulation of *cyclin D3* (*CCND3)* and *suppressor of cytokine signaling 5* (*SOCS5)* through JAK -STAT signaling pathway [56]. In addition, several lncRNAs (XLOC_672329, ALDBGALG0000001429, XLOC_016500, and ALDBGALG0000000253) (Table 1) were predicated to cis-regulate *cholesterol 25-hydroxylase* [*CH25H)*/*cytokine inducible SH2 containing protein* (*CISH)*/*interleukin 1 beta (IL-1β)*/*CD80 molecule (CD80)* to participate in host antiviral responses [56].

The current findings of lncRNA involvement in the infection of ALV are just simply focused on the lncRNA characterization, expression profile, and associated pathways, however, still more proper investigations become necessary.

#### 4.1.3. miRNAs Involved in ALV-Infection

Unlike circRNAs and lncRNAs, many studies have been conducted to explore not only the expression profile but also the underlying functional mechanism of miRNAs involved in ALV infection. miRNA microarray or small RNA sequencing revealed many miRNAs were aberrantly expressed after ALV infection [57,58,59,60]. Among the DEmiRNAs, several of them were verified by qRT-PCR analysis in one or more kinds of ALV-infected materials, including miR-221 [57,58,59,60], miR-222 [57,59,60], miR-1456 [57], miR-1704 [57], miR-1777 [57], miR-1790 [57], miR-2127 [57], let-7b [57,60,61], let-7i [57,60,61], miR-125b [57,58,59,60], miR-375 [57,60], miR-458 [57], miR-193a [58], miR-193b [58,59], miR-148a [59], miR-27b [59], miR-34a [59], miR-130a [59], miR-23b [62] and miR-34b-5p [63]. Obviously, the expression of miR-221/miR-222, let-7b/i, miR-375, miR-125b, and miR-193b were changed in not only one type of materials after ALV-infection, indicating their important roles in response to the ALV.

The qRT-PCR-verified DEmiRNAs were involved in immune-related pathways by analyzing the target genes of the DEmiRNAs. Of which, miR-221/222 may have effect on oncogenicity [57] and exist in several common pathways (MAPK [58], oocyte meiosis [58], Wnt [58], and the chronic myeloid leukemia pathway [61]). In addition, miR-221 was also involved in antigen presentation and apoptosis pathways [59]. miR-1456, miR-1704, miR-1777, miR-1790, and miR-2127 may have effect on oncogenicity [57]. miR-125b was related to the oocyte meiosis and MAPK signaling [58], and may have effect on tumor suppression [57]. miR-193a was involved in Wnt signaling and oocyte meiosis pathway, while miR-193b was included in Wnt signaling and oocyte meiosis pathway [58]. let-7b/i was related to the chronic myeloid leukemia pathway [61]. let-7b/i, miR-375, and miR-458 may be involved in tumor suppression [57].

The underlying mechanisms of some DEmiRNAs involved in ALV infection were revealed (Table 2). For miR-221 and miR-222, they have multiple roles in the regulation of ALV infection. On one hand, miR-221/miR-222 could facilitate DF1 (chicken fibroblast cell line) cell proliferation and migration, and could inhibit the expression of apoptosis-related gene, *BCL-2 modifying factor (BMF)*, leading DF1 cells not easy to undergo apoptosis [64]. On the other hand, miR-221/222 could promote cell proliferation to help ALV replication in DF1 cells. Because the depression of G1/S switch and over proliferation induced by ALV-J for DF1 cells required high expression of miR-221 and miR-221, miR-221 and miR-221 can downregulate the expression of *CDKN1B (cyclin dependent kinase inhibitor 1B)*], which have the ability to arrest the cell cycle and inhibit the cell proliferation process via the *CDKN1B-CDK2 (cyclin dependent kinase 2) /CDK6 (cyclin dependent kinase 6)* pathway [65]. To sum up, miR-221 and miR-222 could promote the ALV progression in DF1 cells through inhibiting apoptosis and facilitating cell proliferation, migration, and growth. In addition, the expression of miR-23b is up-regulated in spleen after ALV-J infection. Moreover, miR-23b could enhance ALV-J replication by regulating *interferon regulatory factor 1 (IRF1)* [62]. Meanwhile, miR-34b-5p is another validated DEmiRNA in spleen after ALV-infection and it could target *melanoma differentiation-associated gene 5 (MDA5)* to promote ALV-J replication by facilitating the cell migration and proliferation [63].

### 4.2. ncRNAs Involved in Marek’s Disease

Marek’s disease virus (MDV) has three serotypes: serotype 1 (MDV-1), serotype 2 (MDV-2), and turkey herpesvirus (HVT) [66]. Only MDV-1 has an oncogenic /pathogenic/tumorigenic effect [48]. Marek’s disease (MD) is caused by MDV. The chickens infected by MDV will cause immunologic suppression, T-cell lymphoma, and neurologic diseases, resulting in tissue or cell damage in chickens. If the MDV susceptible chickens are infected with MDV, the mortality can reach up to 100%, which brings massive loss to the poultry industry [67].

#### 4.2.1. circRNAs Involved in MD

To our knowledge, one circRNA sequencing research has been performed with three types of spleens: First, spleens from MDV infection-induced tumors; second, spleens from the survivors (without any lesion) after MDV infection; third, spleens from non-infected chickens. About 2169 circRNAs were detected in this research, of which, 113 circRNAs were differentially expressed. circRNA-miRNA-mRNA networks showed circZMYM3 could not only interact with 7 miRNAs but also target immune-related genes, such as *SWAP70* and *CCL4*, because *SWAP70* and *CCL4* shared the same target site with miR-214 and circZMYM3. miR-155 was predicted to interact with circGTDC1, circMYO1B, *GATA binding protein 4 (GATA4)*, *peripherin 2 (PRPH2)*, *eomesodermin (EOMES)*, *Rho related BTB domain containing 1 (RHOBTB1)*, and *serine palmitoyltransferase*, *small subunit B (SPTSSB)* [68].

#### 4.2.2. lncRNAs Involved in MD

Chicken MD resistant line 6_3_ and MD susceptible line 7_2_ are model animals for the investigation of responsible biomarkers or clinical diagnosis during the infection of MDV [69]. Expression analysis and bioinformatical functional annotation through RNA sequence by using line 6_3_ and line 7_2_ showed that lncRNAs were aberrantly expressed and were involved in immune-related pathways that indicate that lncRNAs participate in regulating MDV infection [70]. Comparing the expression of the RNAs between infected and noninfected chicken bursa, 425 DElincRNAs and 387 DE genes were found in line 6_3_, while 636 DElincRNAs and 2383 DE genes were identified in line 7_2_. Co-location analysis for the 30 DElincRNAs associated with immune response found that most of the neighboring genes of these lincRNAs were also associated with immune response. Of them, one candidate lincRNA, termed linc-satb1, was positively related to inflammatory/defense/external stimulus response and lymphocyte activation. In addition, the expression of linc-satb1 was correlated with *Special AT-rich binding protein-1 (SATB1)*, which is known to have the ability to regulate chromatin structure and T-cell development/activation [71]. It implies that lnc-satb1 may participate in immune response to MD by regulating *SATB1* [70].

LncRNA could participate in MDV through interacting with both host MDV susceptible/resistant gene. For example, linc-GALMD1 was a DElincRNA of MDV non-infected and infected chickens [72]. *IGLL1 (immunoglobulin lambda-like polypeptide 1)* has distinct expression between line 6_3_ and line 7_2_. It has lower expression in line 6_3_ chickens but higher expression in line 7_2_ chickens after infection of MDV, which implies that *IGLL1* could be a line-specific or susceptible gene in response to MD [72]. Meanwhile, linc-GALMD1 expression level had a positive correlation with *IGLL1* which indicated that linc-GALMD1 could potentially regulate MD by interacting with *IGLL1* [72]. Some lncRNAs were predicted to interact with MD-resistant candidate genes [73]. MSTRG.360.1 was correlated with *C-X-C motif chemokine ligand 12 (CXCL12)*, *TNF receptor superfamily member 6b (TNFRSF6B)*, *SWAP70*, *cytotoxic T-lymphocyte associated protein 4 (CTLA4)*, and *histone deacetylase 9* (*HDAC9*) [73]. MSTRG.6725.1 may interact with *CTLA4* and *joining chain of multimeric IgA and IgM* (*JCHAIN*). MSTRG.6754.1 was associated with *JCHAIN* and *CTLA4* [73]. MSTRG.15539.1 was associated with *SWAP70*, *HDAC9*, *CD72 molecule* (*CD72)*, and *JCHAIN* [73]. MSTRG.7747.5 were strongly correlated with *CD8B molecule (CD8B)*, *HDAC9*, *CD72*, and *Insulin-like growth factor level (IGF-I)* [73].

LncRNA directly regulates MDV gene expression is another approach for chicken host to response to MD. It was evidenced by the upregulation of viral gene-*Meq* (an essential gene for MDV progression), induced by downregulation of linc-GALMD1 [72].

LncRNAs act as an upstream regulator to modulate miRNA expression, thus exhibiting specific function during MD viral infection was also an approach for lncRNA to regulate MDV infection. linc-GALMD3, which has a higher expression level in CD4^+^ T cells after MDV infection, was found to be located on the upstream of the gene transcribes miR-223 [74]. The loss of function of linc-GALMD3 will suppress miR-223 expression and MDV replication. Additionally, DEG analysis of RNA-Seq between the linc-GALMD3 knockdown and control MSB1 (MDCC-MSB1) cells showed that about 27 DEGs were also miR-223 target genes, which implied the *cis* regulatory mechanism of linc-GALMD3 on miR-223 [74]. The linc-GALMD3-miR-223-target DEGs interaction network could be used for further investigation during MDV infection.

The selected lncRNAs, their targets, and their functions during the MDV infections were shown in Table 3.

#### 4.2.3. miRNAs Involved in MD

Numerous researches have been focused on the exploration of miRNA expression profile and their potential role during the infection of MDV. Hundreds of miRNAs were significantly differentially expressed between MDV-infected and noninfected groups [75,76,77,78,79,80]. let7 [75], miR-199a-1 [75,77], miR-26a [75,77], miR-181a [75,77] miR-16 [75] miR-223 [76], miR-150 [76], miR-155 [76,79], miR-221 [77], miR-199 [77], miR-15b [78], miR-456 [78], let7i [78], miR-762 [79], miR-29b [79], miR-140-3p [81], miR-199-3p [81], and miR-221-5p [81], were verified by qRT-PCR/Northern blot analysis.

The classical functional mechanism of miRNAs in regulating MDV infection is targeting the 3′UTR of their target genes and inhibiting the transcription of the mRNA or interfering their translation [82]. miR-221/miR-222 were significantly upregulated after MDV infection. The *cyclin-dependent kinase (cdk) inhibitors 27 ^Kip1^* has direct effect on cell cycle and cell proliferation [82]. Conserved binding site between miR-221/miR-222 and 27 ^Kip1^ was verified in several species and their target relationship has been confirmed in several cancer cells [82]. In avian, miR-221/miR-222 would also target *27 ^Kip1^* to regulate MSB1 cell proliferation [83]. *Retinoid Acid Receptor-Related Orphan Receptor Alpha* (*RORA*) is a suppressor for tumor and could be involved in immunity, inflammation metabolism [84], and tumor initiation [85]. In MBS1 cells, miR-155 could increase the proliferation, invasiveness, and reduce apoptosis by targeting *RORA* [86]. Target interactions of miR-181a with *MYB proto-oncogene like 1 (MYBL1)*/ *insulin like growth factor 2 mRNA binding protein 3 (IGF2BP3)*, and miR-26a with *eukaryotic translation initiation factor 3 subunit A* (*EIF3A)*, were confirmed by luciferase reporter assays [77]. Furthermore, miR-181a was verified to inhibit proliferation of MSB1 by targeting *MYBL1* [87]. *Never in mitosis gene A (NIMA)-related kinase 6* (*NEK6*) is another verified target gene of miR-26a and showed opposite expression pattern with miR-26a between MDV-infected spleens and noninfected controls. miR-26a would exhibit suppression to MSB1 cell proliferation through inhibiting *NEK6* [88]. miR-103-3p decreases cell migration by targeting *transcription factor Dp-2 (E2F dimerization partner 2)* (*TFDP2*) and *cyclin E1* (*CCNE1*) [89]. For miR-130a, it could inhibit MSB1 cell proliferation and migration by targeting *homeobox A3* (*HOXA3*) and *MyoD family inhibitor domain containing* (*MDFIC*) [90]. miR-219b could target *B-cell chronic lymphocytic /lymphoma 11B* (*BCL11B*) and suppresses the proliferation, migration, and invasion of MSB1 cells [91].

As a homologous to MDV-encoded miRNA to participate in regulate the process of MDV infection is another miRNA regulatory mechanism in avian. This was discovered for miR-155, miR-29b, and miR-221. miR-155 shared seed sequence with the same target genes with MDV-induced miRNA, MDV-1-miR-M4 [92]. The shared target genes including *PU.1*, *HIVEP2 (HIVEP zinc finger 2)*, *CEBPβ (CCAAT enhancer binding protein beta)*, *PDCD6 (programmed cell death 6)*, *BCL2L13 (BCL2 like 13)*, *RREB1 (ras responsive element binding protein 1)*, *GPM6B (glycoprotein M6B)*, *MAP3K7IP2 (TGF-beta activated kinase 1/MAP3K7 binding protein 2)*, and *c-Myb (MYB proto-oncogene*, *transcription factor)* [93,94]. Many of these genes are known to have a specific role in regulating tumor cell proliferation/apoptosis and tumor formation. Additionally, MDV-1-miR-M4 is essential for MDV infection due to the deletion of MDV-1-miR-M4 or a 2 nucleotides mutation on its seed region will lead to an inhibition for the induction of lymphomas. This inhibition could be rescued by gga-miR-155, thus in turn implied miR-155 is a crucial regulator for lymphomas. *Toll-like receptor 3 (TLR3)* is crucial for innate and adaptive immunity. It was abundantly expressed in MDV-infected CEF cells, resulting in replication inhibition of the RB1B strain of MDV. However, such inhibition would alter both MDV1-miR-M4-5p and miR-155 [95]. miR-29b shared a seed sequence with MDV2-miR-M21, and expression of MDV2-miR-M21 may ensure viral proliferation in host cells. miR-221 shared a seed sequence with both hvt-miR-H14-3p and mdv1-miR-M32 [92,96,97]. As the homologous miRNAs of virus-encoded miRNAs, some host miRNAs share the same biding site and even same target genes with virus-encoded miRNAs to influence the biological function of virus-encoded miRNAs may be a new strategy to resist virus invasion.

The interaction of host miRNAs with virus-encoded gene is also a regulatory mechanism for miRNAs during the virus infection. For instance, the viral oncoprotein *Meq* could regulate the expression of miR-21 by binding to the promoter region of miR-21. Meanwhile, miR-21 could target *chicken programmed death cell 4* (*PDCD4*) to suppress growth and apoptosis escape of tumor cells [98]. It provided a viral gene-host miRNA-host gene regulation mechanism involved in the MDV infection. Meanwhile, miR-155 was found to target viral env transcripts and could significantly lower the env transcripts abundance in MSB1 and CEF cells [99].

The selected-verified miRNAs and their target genes and their functions during the MDV infections are presented in Table 4.

### 4.3. ncRNAs Involved in Infectious Bursal Disease

Infectious bursal disease (IBD) is a virus disease of young chickens which is caused by avian infectious bursal disease virus (IBDV). IBDV has negative effective on T cells proliferation, causing immunologic suppression in chicken [100,101]. Moreover, IBD has caused death and vaccination failure to other disease in chicks [101].

#### 4.3.1. LncRNAs Involved in IBD

Dendritic cells (DCs) have special role in both innate and acquired immune during virus infection [102]. A microarray study on IBDV-stimulated DCs and non-stimulated DCs revealed 114 lncRNAs, 18 miRNAs, and 965 mRNAs were differentially expressed after stimulated with IBDV [103]. Functional annotation of the DElncRNAs, DEmiRNAs, and DE genes showed that there was an association with cellular response to starvation, protein localization, the RNA biosynthetic process, etc. These were involved in JAK-STAT/MAPK/mTOR/neurotrophin/CCR5/Interleukin-17 (IL-17) signaling pathways, as predicted by a pathway analysis [103].

#### 4.3.2. miRNAs Involved in IBD

miRNAs could regulate viral infection through regulating the IBDV genome sequence. IBDV as a double stranded virus has two segments (segment A and B) and encodes five viral proteins, VP1-VP5, which play different roles in the infection process of IBDV to the host. Of them, *VP1* is required for both IBDV replication and virulence [104]. As the target of tumor suppressors in human [105,106], miR-21 could suppress IBDV replication via inhibiting the expression of *VP1* in avian [107]. Similarly, miR-454 targeting IBDV genomic segment B to inhibit IBDV replication [108]. miR-130b could inhibit IBDV replication through targeting IBDV segment A [109].

Apart from targeting the viral genome sequence, miRNA could also regulate the IBDV infection through targeting regulators of *type I interferon (IFN)*, as *IFN* is a crucial cellular molecule to combat viral infection and interferon regulatory factor-dependent pathways play important roles during the infection of various disease. *SOCSs (Suppressors of Cytokine Signaling)* gene family was known as one kind of negative regulators of *IFN*. Specially, the inhibition of *SOCS6* can increase the expression of *IFN-β*. miR-454 could suppress the IBDV replication through suppression of *SOCS6* [108]. Similarly, miR-130b could facilitate *IFN-β* expression through binding to the host *SOCS5* to inhibit IBDV replication [109]. For miR-155, it was found to suppress IBDV replication through targeting not only *SOCS1* but also another type I IFN negative regulator, *TANK (TNF receptor-associated factor family member-associated NF-κB activator)* [110]. On the contrary, miR-9 could inhibit *IFN* expression by inhibiting *IRF2* and then promoting the replication of IBDV [111]. miR-142-5p was founded to decrease the activity of the *IFN-β* by directly by targeting the *MDA5* and then promoting IBDV replication through IRF7 (interferon regulatory factor 7) signaling pathway [112]. Additionally, miR-2127 has been found to promote IBDV replication by suppressing *p53* translation and attenuating *p53*-mediated innate immune response against IBDV infection [113].

More novelty, IBDV alters the expression of responsible miRNAs through inducing the demethylation of the miRNA promoter is a new strategy for IBDV to protect themselves for survival from the immune responses induced by related-miRNAs. For instance, IBDV infection could induce the promoters of pre-miR-27 and pre-miR-16-2 demethylation, thus upregulating miR-27b-3p and miR-16-5p expression [114,115]. Furthermore, miR-27b-3p could increase the expression of chicken IFN-β, NF-κB, and IRF3 (interferon regulatory factor 3) by targeting SOCS3 and SOCS6, thus inhibiting IBDV replication [114]. miR-16-5p increased the caspase-9/3 activity through targeting Bcl2, thus promoting the IBDV-induced apoptosis [115].

### 4.4. ncRNAs Involved in Infectious Bronchitis Virus (IBV) Infection

Avian infectious bronchitis virus (IBV) belongs to coronavirus. It mainly replicates in chicken epithelial cells and could infect both meat type and commercial type of small or old chickens [116,117]. The investigation of the host responsible mRNAs and lncRNAs in IBV-infected DCs revealed thousands of mRNAs were differentiated expressed in IBV-infected avian DCs and noninfected cells [118]. With 1093 up-regulated and 845 down-regulated. miRNAs and lncRNAs interaction provided further information for the candidate therapeutic biomarkers in response to IBV infection [118]. miRNA transcriptome analysis in chicken kidneys showed 58 DE miRNAs were found and were shown to be mostly associated with immune response, catalytic activities, metabolic processes, and gene expression [119]. Among them, miR-1723, miR-7b, miR-222b-3p, miR-1782, miR-6516-3p, miR-202-5p, miR-1559-3p, miR-449a, miR-1454, and miR-1563 were verified by qRT-PCR [119]. As a predicted DEmiRNA by sequence analysis, miR-146a-5p was also found to promote IBV replication of by targeting *tumor necrosis factor receptor superfamily member 18* (*TNFRSF18*) and *IL-1 receptor associated kinase-2 (IRAK2)* [120]. As another DEmiRNA, miR-30d was showed to inhibit IBV replication through suppressing *ubiquitin-specific protease 47* (*USP47*) in HD11 (avian macrophage-like cells) cells [121].

### 4.5. ncRNAs and Newcastle Disease (ND)

Newcastle disease is caused by a single-stranded RNA virus, Newcastle disease virus (NDV), resulting in a high mortality rate in infected chicken worldwide [122].

Only few studies have been focused on ncRNA investigation associated with ND. One study performed mRNA-seq and small RNA-seq to identify the interaction pairs of mRNA-miRNA involved in ND. 1069 miRNA-mRNA pairs were found. Among them, the interaction of miR-203a and *transglutaminase 2 (TGM2)* were confirmed by both qRT-PCR and dual-luciferase reporter assay [123]. Additionally, miR-19b-3p could promote the expression of inflammatory cytokines and suppress NDV replication by targeting *ring finger protein 11 (RNF11)* and *zinc-finger protein*, *MYND-type containing 11* (*ZMYND11*), which are two negative moderators of NF-κB signaling [124]. miR-455-5p could suppress NDV replication by directly targeting *SOCS3* [125]. miR-375 inhibited NDV growth through targeting the *M gene* of NDV and host *Drosophila-like RNA binding protein 4* (*ELAVL4)* [126].

### 4.6. ncRNAs and Avian Influenza

Avian influenza (AIV) is caused by avian influenza type A viruses, a virus belongs to *Orthomyxoviridae* family [127]. Other viruses in this family include influenza B and influenza C virus, but only influenza A virus has been found in birds [127,128]. Although avian influenza causes only mild illness, it still can be very dangerous because of its high transmission among birds, and even among human beings [129,130].

Several RNA-seq researches had revealed the expression profile and associated pathways in response to AIV infection in different tissues and cells in avian [131,132,133,134]. According to the bioinformatics analysis of the expression and the associated pathways of the DEmiRNAs in those researches, miR-34a, miR-122-2, miR-122-1, miR-206, miR-146a, miR-155, miR-1719, miR-1599, miR-1594, miR-451, miR-146, miR-1576, miR-1636, miR-206, miR-142-5p, miR-17-5p, miR-19b miR-133c, miR-1710, miR-181a/b, miR-30b/c/e, and miR-455 were considered to be strong candidate miRNAs related to the immune response of AIV infection in chickens [131,132,133,134].

The genome of type A influenza includes eight segments and each segment-encoded protein is crucial for viral replication and pathogenesis [133]. miRNAs interact with virus genes is also one approach for miRNA to control or resist AIV. Many chicken host miRNAs have been analyzed to have target relationship with avian influenza [135]. Specially, the non-structural protein 1 (NS1) is one protein of segment 8 of H5N1 genome that is directly associated with the pathogenicity of the influenza strain. miR-1658 could regulate the AIV infection by targeting the *NS1* gene of H5N1 genome [135]. Moreover, *PB1 (polymerase PB1)*, *PB1-F2 (PB1-F2 protein)*, and *N40* were three proteins encoded by segment 2 of H5N1 virus. miR-1710, miR-133c, and miR-146c were predicted to target PB1-F2, PB1, and N40 proteins, implying that they potentially regulate H5N1 infecting by interacting with virus genes [133].

### 4.7. ncRNAs and Reticuloendotheliosis (RE)

Reticuloendotheliosis, a reticuloendotheliosis virus (REV)-induced disease, is highly prevalent in avian and human [136]. It can cause chronic B cell and T-cell lymphomas [137], delayed growth, immunodepression, and tumor in birds [138].

To the best of our knowledge, only few studies related to ncRNAs have been investigated during RE infections. High-throughput sequencing has been conducted in REV-infected and non-infected tissues or cells and the results identified many candidate DEmiRNAs involved in REV infection. These DEmiRNAs could target immune-related genes and participate in immune-related pathways [138,139,140]. Among them, miR-2945, miR-106-3p/5p, miR-29b/3p/, miR-7b, miR-16c-5p/, miR-122-5p, miR-155, miR-18a-5p, miR-147, miR-184-3p, miR-222b-5p/3p, miR-145b-5p, miR-20b-5p, and miR-1b-3p expressions were verified by qRT-PCR. GO term analysis showed that these miRNAs are related to apoptotic process, intracellular signal transduction, cell death regulation. Also, many important KEGG pathways were enriched, such as MAPK signaling, endocytosis, focal adhesion, apoptosis, cell cycle pathways, mTOR signaling, cell growth and death, and immune system.

Specially, of so many DEmiRNAs, the underlying regulatory mechanisms of miR-155 and novel-72 were verified and indicated that they play distinct role in response REV infection. For miR-155, it showed a high expression level during REV infection in CEF cells. It has been demonstrated that miR-155 could inhibit apoptosis by targeting *caspase-6* and accelerate cell cycle through inhibiting *FOXO3a* and *JARID2 (jumonji*, *AT rich interactive domain 2)* expression during the REV infection [141,142]. For novel-72, it showed a significantly lower expression in REV-infected cells. Four predicated target genes of novel-72, *PDPK1 (phosphoinositide dependent protein kinase-1)*, *mTOR (mammalian target of rapamycin)*, *S6K1 (S6 Kinase 1)*, and *eIF4E (Eukaryotic Initiation Factor 4E)* of mTOR signaling pathway were related to cell proliferation and survival, indicating that novel-72 could be involved in mTOR signaling pathway to mediate the cell fate during REV infection [139].

The selected-verified miRNAs and their target genes and their functions during the IBDV, IBD, NDV, AIV, and REV infections were listed below (Table 5).

**Figure 2:**
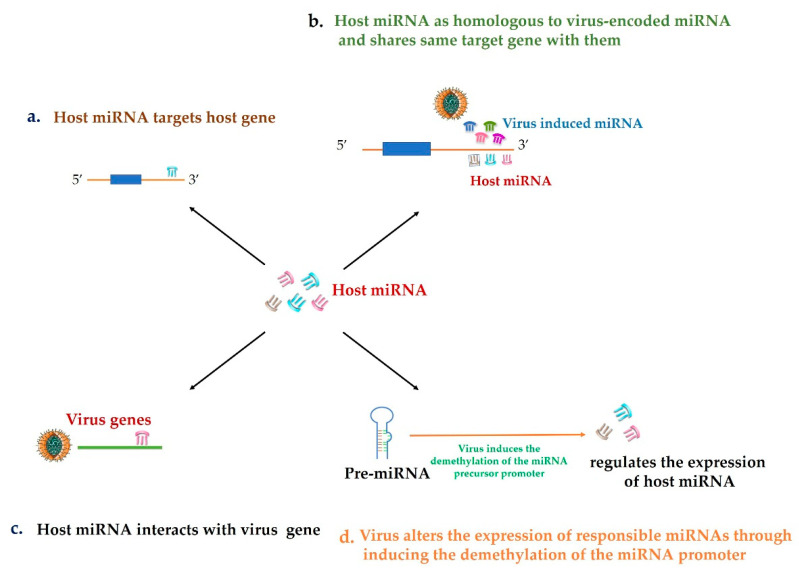
A model of host miRNA participated in regulating virus infection through four functional mechanisms. (**a**) Host miRNA directly targets the host gene. (**b**) Host miRNA as homologous to virus-encoded miRNA and shares same target gene with them. (**c**) Host miRNA interacts with virus genes. (**d**) Virus induce the pre-miRNA demethylation to regulate the expression of virus responsible miRNA.

As avian virus disease has been well studied, the mechanisms that miRNAs participate in avian virus diseases are clear now. There are four functional mechanisms have been reported: (1) Direct interaction with the host gene (Figure 2a) [82]; (2) acts as homologous to virus-encoded miRNAs and shares the same target gene with virus-encoded miRNAs (Figure 2b) [92,93,94,95,96,97,143]; (3) interacts with virus genomic gene (Figure 2c) [98,99,107,108,109]; (4) alters the expression of responsible miRNAs through inducing the demethylation of the miRNA promoter to protect virus themselves to survival from the immune responses induced by related-miRNAs (Figure 2d) [114,115].

## 5. ncRNAs Related to Parasitic Infection

### 5.1. ncRNAs Associated with Coccidiosis and Cryptosporidium Baileyi Infection

Chicken coccidiosis is an epidemic parasitic disease can be caused by nine *Eimeria* spices, including *Eimeria tenella*, *necatrix*, *maxima*, *acervuline*, *brunetti*, *mivati*, *hagani*, *praecox*, and *mitis*. They are infecting chicken intestinal epithelial cells. Among them, *Eimeria tenella*, *Eimeria maxima*, and *Eimeria necatrix* have the higher pathogenicity. One RNA sequence analysis of differentially expressed lncRNAs, circRNAs, and mRNAs has been performed in chicken small intestines during *Eimeria necatrix* infection. This RNA sequence analysis obtained 1543 DEmRNAs, 95 DElncRNAs, 13 DEcircRNAs [144]. Functional annotation for lncRNAs, miRNAs, and circRNAs were correlated with chicken host immune defense and pathogenesis during *E. necatrix* infection [144]. In addition, miRNA expression was compared between naturally or *Eimeria maxima* and *Eimeria acervulina* -infected Ross 308 broilers using RNA sequencing. Among DEmiRNAs, miR-122-5p, miR-144-3p, and miR-205b were verified by qRT-PCR [145], however, their potential roles during avian coccidiosis need further investigations. The regulatory mechanisms of ncRNAs in response to coccidiosis remain largely unknow.

### 5.2. ncRNAs Associated with Cryptosporidium Baileyi Infection

*Cryptosporidium baileyi* (*C. baileyi*) belongs to *Cryptosporidium* species in birds [146]. It can infect in the epithelial cells of bursa or respiratory tract, causing respiratory diseases [146,147]. In broiler chickens, it can cause high mortality and morbidity [148]. One research focused on the identification of lncRNAs, circRNAs, and mRNAs involved in *C. baileyi* infection in chickens’ tracheal tissues. It found 124 lncRNAs, 104 circRNAs, and 1317 mRNAs were differentially expressed. GO analysis found that the targets and source genes of mRNAs and lncRNAs are all involved in immune response and immune system process, while KEGG analysis showed that they are related to intestinal immune network for IgA production, cell adhesion molecules (CAMs), cell cycle, and the cytokine-cytokine receptor interaction. The circRNAs were significantly enriched in pathways associated with tight junction and glycerolipid metabolism, nucleotide sugar metabolism, and amino sugar [149]. However, the underlying molecular mechanism of ncRNAs in response to *C. baileyi* infection needs further investigation.

## 6. ncRNAs Related to Bacterial Infection

### 6.1. ncRNAs Involved in Campylobacter Jejuni (C. jejuni)

*Campylobacter jejuni* (*C. jejuni*) is a foodborne pathogen that causes human diarrhea on consuming chicken products which are contaminated by *C. jejuni* [150]. Two studies on ncRNAs associated with *C. jejuni* have been reported [138,151,152]. Solexa sequencing for cecal tissue from *C. jejuni* inoculated and non-inoculated SPF chicken showed 4 miRNAs, miR-155, miR-1416-5p, miR-19b-3p, and miR-19a-3p were significantly differentially expressed [151]. In addition, miR-30b, miR-30c, miR-148a, and miR-1416–5p were able to interact with the *SOCS3* in response to *C. jejuni* inoculation which indicated they may mediate *C. jejuni* infection through regulating *SOCS3* [152].

### 6.2. ncRNAs and Necrotic Enteritis (NE)

Necrotic enteritis is usually caused by *Clostridium perfringens* with symptoms of weight depression, appetite loss, and even cause death to chickens [153]. Small RNA-seq showed many miRNAs were differently expressed after challenged by NE [154,155,156]. miR-215, miR-194, miR-217, miR-200a, miR-216a, miR-200b, miR-216b, miR-34b, miR-429, miR-1684, miR-9-5p, miR-196-5p, miR-20b-5p and let-7d were confirmed by qRT-PCR [154,156]. Some miRNAs were correlated with immune-related mRNA expression levels, such as miR-216 and *TGFβR2 (transforming growth factor beta receptor 2)*, miR-30b/miR-30c/miR-455–5p and *SOCS3*, miR-181a/b and *CXCL14 (C-X-C motif chemokine ligand 14)*, miR-429 and *TNFSF11β (TNF superfamily member 11 beta)*, miR-223 and *HSP90β1 (heat shock protein 90 beta family member 1)*, miR-1329 and *NFKBIZ (NFKB inhibitor zeta)*, miR-1674 and *ARHGEF (FERM*, *ARH/RhoGEF and pleckstrin domain protein 1)*, miR-30e/ miR-32 and *SERPINF1 (serpin family F member 1)* [155]. Many immune-related pathways were identified for DEmiRNA targets, such as MAPK, ErbB, Notch, TGF-*β*, JAK-STAT, Cytosolic etc. Specially, JAK-STAT as the key pathway, 20 genes of this pathway showed marked differential expression, strongly suggesting the crucial role of JAK-STAT pathway in regulating NE [157]. The miR-200a-3p could regulate inflammatory factor and MAPK signaling pathway-related gene in response to NE. For instance, it regulated the expression of *IL-1β*, *IFN-γ*, *IL-12p40 (interleukin 12B)*, *IL-17A (interleukin 17A)*, *LITAF (lipopolysaccharide induced TNF factor)*, and *ZAK (also known as MAP3K20- mitogen-activated protein kinase kinase kinase 20)*, *MAP2K4 (mitogen-activated protein kinase kinase 4)*, and *TGFβ2 (transforming growth factor beta 2)* of MAPK signaling pathway [158]. The miR-10a could inhibit the expression of MyD88-dependent pathway-related genes, including *TRAF6 (TNF receptor associated factor 6)*, *TAK1 (TGF-beta activated kinase 1 (MAP3K7) binding protein 1)*, *NF-κB1 (nuclear factor kappa B subunit 1)*, and downstream genes of the MyD88-dependent pathway related genes, including *IL-1β*, *IFN-γ*, *IL-12p40*, *TNFSF15 (TNF superfamily member 15)*, and *LITAF*. It strongly indicated that miR-10a regulates the NE through Toll-like receptor pathway [159].

### 6.3. ncRNAs and Salmonella enterica Serovar Enteritidis Infection

*Salmonella enterica serovar enteritidis* (SE) is the most common serotype of the Salmonella bacteria. Chicken SE infection is mainly caused by ingesting water or consuming food contaminated with *Salmonella bacteria*. CircRNAs have been identified through next-generation sequencing during SE infection [160]. Total of 62 circRNAs were significantly differentially expressed (30 upregulated and 32 downregulated) between the control and treated groups. The immune-related pathway associated with the DE circRNAs including, adrenergic signaling in cardiomyocytes, herpes simplex infection, and signaling pathway of MAPK/ p53/VEGF/Notch. Three immune-related genes, *TXNDC9 (thioredoxin domain containing 9)*, *JAG2 (jagged canonical Notch ligand 2)*, and *NFATC2 (nuclear factor of activated T cells 2)*, generated 11 DE circRNAs involved in the B cell receptor signaling pathway, B cell proliferation, drug biological process, and cytokine production [160]. In addition, miR-1306-5p regulated the immune response to SE by inhibiting the *Toll-interacting protein (Tollip)*, which is an up-regulator for inflammatory cytokines- *NF-κB*, *IL-6 (interleukin 6)*, *TNF-α*, and *IL-1β (interleukin 1-beta)* [161]. miR-101-3p and miR-155 could alter the expression of their target genes, *LRRC59 (leucine rich repeat containing 59)* and *IRF4 (interferon regulatory factor 4)*, and decrease the expression of pro-inflammatory cytokines during SE infection [162]. In addition, miR-1662, miR-1416-5p, and miR-34a-5p showed opposite expression pattern with the immune-related target genes of *BCL10 (B-cell CLL/lymphoma 10)*, *TLR21 (Toll-like receptor 21)*, *NOTCH2 (notch 2)*, *TLR1LA (toll-like receptor 1 family member A)*, and *THBS1 (thrombospondin 1)*, indicating their important roles in SE infection [163].

### 6.4. ncRNAs and Salmonella typhimurium

*Salmonella typhimurium (S. typhimurium)* commonly occurred in avian and humans [164]. It is a major food-borne pathogen and has an impact on the microbiological safety of eggs which is also a risk factor to humans [165]. A previous study revealed 14 miRNAs that were significantly altered by the infection of *S. typhimurium* [166]. Among them, miR-3525, miR-215-5p, miR-193a-5p, miR-122-5p, and miR-375 were verified by qRT-PCR. The predicted DEmiRNAs target genes were enriched in immune system development, stress-activated MAPK cascade, the regulation of cAMP-dependent protein kinase activity, and the regulation of immune system process (such as, MAPK and Wnt signaling pathways) [166].

The selected-verified miRNAs and their target genes and their functions during the bacterial infections were listed below (Table 6).

## 7. Concluding Remarks

ncRNAs are recognized as powerful regulatory molecules and have been largely investigated in various disease process of different species, including avians. The unique functional mechanisms of miRNA, lncRNA, and circRNA were revealed.

Although the underlying functional mechanism of ncRNAs has been revealed in many species, it is still beginning to emerge in response to avian disease, except for miRNAs. Researches on circRNAs and lncRNAs are basically performed on the analysis of their expression profile and associated pathways. ncRNAs have been found to play roles during the infection of various avian diseases through different approaches. For lncRNAs and circRNAs, they mainly exhibit their function through lncRNA/circRNA-miRNA-mRNA axis, however, in some cases they could also regulate the immune process by directly interacting with RNA binding proteins or virus gene sequence.

miRNAs can regulate various aspects of immune response process. In avian, some specific miRNAs were identified to be differentially expressed in many different types of chicken diseases from virus, parasite, and bacteria, such as miR-155 [68,110,141,142], miR-221/222 [60,83,119,138], miR-130 [59,90,109], miR-30 [121,152,155], miR-34 [59,63,132,154,163], let-7 [57,81,155], miR-181 [77,134,155], miR-16 [80,115], miR-455 [125,134,155], etc. It indicated their extensive roles in regulating avian immunity. Especially, miR-155 has been reported to play important roles in response to different kinds of avian diseases, such as ALV [68], MD [76], IBD [110], Avian influenza [132], Reticuloendotheliosis [141,142], *Campylobacter jejuni* [151], and also *Salmonella enterica* serovar Enteritidis [162].

The functional mechanisms of ncRNAs in regulating avian immune system can be categorized into three mechanisms: First, ncRNAs interact with pathogenic gene of virus/parasite/bacterium; second, ncRNAs act as a homologous to the product of pathogen; third, ncRNAs directly interact with host resistant and susceptible genes.

## Figures and Tables

**Table 1:** Selected lncRNAs and their potential function during the Avian leukosis virus (ALV) infection.

lncRNA	Target	Function	Ref
XLOC_672329	*CH25H*	participate in host antiviral responses	[56]
ALDBGALG0000001429	*CISH*	participate in host antiviral responses	[56]
XLOC_016500	*IL-1β*	participate in host antiviral responses	[56]
ALDBGALG0000000253	*CD80*	participate in host antiviral responses	[56]

**Table 2:** Selected-verified miRNAs and their function during the ALV infection.

miRNA	Target Gene	Function	Refs
miR-23b	*IRF1*	enhance ALV-J replication	[62]
miR-34b-5p	*MDA5*	promote ALV-J replication	[63]
miR-221/miR-222	*BMF*, *CDKN1B*	inhibit apoptosis and facilitating cell proliferation/migration/growth	[64,65]

**Table 3:** Selected lncRNAs and their potential function during the Marek’s disease virus (MDV) infection.

LncRNA	Target	Function	Ref
linc-GALMD1	*IGLL1*, *Meq*	regulate MDV infection	[72]
MSTRG.360.1	*CXCL12*, *TNFRSF6B*, *SWAP70*, *CTLA4*, *HDAC9*	related to MD-resistance	[73]
MSTRG.6725.1	*CTLA4*, *JCHAIN*	related to MD-resistance	[73]
MSTRG.6754.1	*CTLA4*, *JCHAIN*	related to MD-resistance	[73]
MSTRG.15539.1	*SWAP70*, *HDAC9*, *CD72 JCHAIN*	related to MD-resistance	[73]
MSTRG.7747.5	*CD8B*, *HDAC9*, *CD72*, *IGF-I*	related to MD-resistance	[73]
linc-GALMD3	*miR-223*	involved in MDV replication	[74]

**Table 4:** Selected-verified miRNAs and their function during the MDV infection.

miRNA	Target Gene	Function	Refs
miR-221/miR-222	*27 ^Kip1^*	regulate MSB1 cell proliferation	[83]
miR-155	*RORA*	increase proliferation, invasiveness and reduce apoptosis	[86]
miR-181a	*MYBL1*, *IGF2BP3*	inhibit MSB1 cell proliferation	[77,87]
miR-26a	*EIF3A*, *NEK6*	inhibit MSB1 cell proliferation	[77,88]
miR-103-3p	*CCNE1*, *TFDP2*	inhibit MSB1 cell migration	[89]
miR-130a	*HOXA3*, *MDFIC*	inhibit MSB1 cell proliferation and migration	[90]
miR-219b	*BCL11B*	suppresses proliferation, migration and invasion of MSB1 cell	[91]
miR-21	*PDCD4*	suppress growth and apoptosis of tumor cells	[99]

**Table 5:** Selected-verified miRNAs and their function during the infectious bursal disease virus (IBDV), IBV, Newcastle disease virus (NDV), AIV, and reticuloendotheliosis virus (REV) infections.

miRNA	Target Gene	Function	Refs
miR-21	*VP1*	suppress IBDV replication	[107]
miR-454	*SOCS6*, IBDV genomic segment B	inhibit IBDV replication	[108]
miR-130b	SOCS5	inhibit IBDV replication	[109]
miR-155	*SOCS1*, *TANK*	suppress IBDV replication	[110]
miR-9	*IRF2*	promote IBDV replication	[111]
miR-142-5p	*MDA5*	promote IBDV replication	[112]
miR-2127	*p53*	promote IBDV replication	[113]
miR-27b-3p	*SOCS3*, *SOCS6*	inhibit IBDV replication	[114]
miR-16-5p	*Bcl2*	promote IBDV-induced apoptosis	[115]
miR-146a-5p	*TNFRSF18*, *IRAK2*	promote IBV replication	[120]
miR-30d	*USP47*	inhibit IBV replication	[121]
miR-203a	*TGM2*	unknow	[123]
miR-19b-3p	*RNF11*, *ZMYND11*	suppress NDV replication	[124]
miR-455-5p	*SOCS3*	suppress NDV replication	[125]
miR-375	*M* gene of NDV, *ELAVL4*	inhibit NDV growth	[126]
miR-1658	*NS1* gene of H5N1 genome	regulate AIV infection	[135]
miR-155	*FOXO3a*, *JARID2*	inhibit apoptosis and accelerate cell cycle during the REV infection	[141,142]

**Table 6:** Selected miRNAs and their potential function during the bacterial infections.

miRNA	Target Gee	Function	Ref
miR-30b, miR-30c, miR-148a, and miR-1416–5p	*SOCS3*	in response to *C. jejuni* inoculation	[152]
miR-216	*TGFβR2*	in response to NE	[155]
miR-30b/miR-30c/miR-455–5p	*SOCS3*	in response to NE	[155]
miR-181a/b	*CXCL14*	in response to NE	[155]
miR-429	*TNFSF11β*	in response to NE	[155]
miR-223	*HSP90β1*	in response to NE	[155]
miR-1329	*NFKBIZ*	in response to NE	[155]
miR-1674	*ARHGEF*	in response to NE	[155]
miR-30e/ miR-32	*SERPINF1*	in response to NE	[155]
miR-1306-5p	*Tollip*	regulated the immune response to SE	[161]
miR-101-3p, miR-155	*LRRC59*, *IRF4*	decrease the expression of pro-inflammatory cytokines during SE infection	[162]
